# Supplementary material for: A longitudinal analysis of COVID-19 lockdown stringency on sleep and resting heart rate measures across 20 countries
Source: Sci Rep. 2021 Jul 13;11:14413. doi: 10.1038/s41598-021-93924-z (PMC8277902; doi:10.1038/s41598-021-93924-z)
Supplement: Supplementary file 2 — Supplementary Information 2. [file 41598_2021_93924_MOESM2_ESM.html]

Supplementary Materials 2


# Supplementary Materials 2

Last Updated: 07 May, 2021

### Contents:

1. Figure 1a. Midsleep Time  
    1.1. Midsleep Time for Individual Countries
2. Figure 1b. Midsleep Variability  
    2.1. Midsleep Variability for Individual Countries
3. Figure 1c. Resting Heart Rate  
    3.1. Resting Heart Rate for Individual Countries

**NOTE**

- Dates in 2019 are shifted one day earlier to allow better and easier comparison to dates in 2020. E.g, 2019 Jan 02 was set as 2020 Jan 01
- When hovering on the plots, there are functions (e.g. zooming, panning, resetting) on the top right corner.

  - Click on specific years in the legend to unselect specific years for better viewing
  - Zoom in to have a finer-grained view
  - Reset axes to revert to original view

## 1. Figure 1a. Midsleep Time

### 1.1. Midsleep Time for Individual Countries

Use the dropdown menu to select individual countries. When hovering on the plot, there are functions (e.g. zooming, panning, resetting) on the top right corner.

- Click on specific years in the legend to unselect specific years for better viewing
- Zoom in to have a finer-grained view
- Hover over any point to view specific values of the measure and weekday/weekend status
- Reset axes to revert to original view

## 2. Figure 1b. Midsleep Variability

### 2.1. Midsleep Variability for Individual Countries

## 3. Figure 1c. Resting Heart Rate

### 3.1. Resting Heart Rate for Individual Countries
